# Minimally invasive percutaneous osteosynthesis versus ORIF for Sanders type II and III calcaneal fractures: a prospective, randomized intervention trial

**DOI:** 10.1186/s13018-017-0511-5

**Published:** 2017-01-18

**Authors:** Cong Jin, Dong Weng, Wanlei Yang, Wei He, Wengqing Liang, Yu Qian

**Affiliations:** Department of Orthopaedics, Shaoxing People’s Hospital, Zhongxing North Road, Shaoxing, Zhejiang 312000 People’s Republic of China

**Keywords:** Minimally invasive, Calcaneal fractures

## Abstract

**Background:**

This randomized controlled trial compared the clinical outcomes and complications of a novel minimally invasive percutaneous osteosynthesis (MIPO) with those of conventional treatment via an extended L-shaped lateral approach for calcaneal fractures.

**Methods:**

Sixty-four patients with displaced intraarticular calcaneal fractures were enrolled. The patients were randomly allocated to receive either MIPO (29 patients) or open reduction and internal fixation via an extended L-shaped lateral approach (35 patients). The same calcaneal plate (AO Synthes, Oberdorf, Switzerland) was used in both groups. The primary clinical outcomes included operative time, VAS postoperatively, and wound healing complications. Secondary clinical outcomes included time to operation, length of incision, postoperative drainage, length of hospital stay, medical expense, AOFAS score, and SF-36 score. Preoperative and postoperative calcaneal height, width, and length, Bohler’s angle, and Gissane’s angle were compared.

**Results:**

The operative time in the MIPO group was 52.5 ± 11.1 min, which was significantly shorter than 82.8 ± 16.2 min in the conventional treatment group (*P* < 0.001). One week postoperatively, the VAS value was 3.2 ± 1.4 in the MIPO group, which was lower than that in the conventional treatment group, 3.9 ± 1.3 (*P* = 0.038). In the conventional treatment group, 13 of 35 fractures (37.1%) had wound healing problems, whereas this issue occurred in only 2 of 29 fractures (6.7%) in the MIPO group (*P* = 0.004). In the MIPO group, deep and superficial infections occurred in none of the cases and 1 of 29 (3.4%) patients, respectively. Length of incision in the MIPO group was shorter than that in the conventional treatment group (4.2 ± 0.6 vs. 10.9 ± 1.5 cm; *P* < 0.001). Hospital stay was 9.7 ± 2.8 days in the MIPO group and 11.7 ± 2.6 days in the conventional treatment group (*P* = 0.004). At the last follow-up, the SF-36 scores and AOFAS scores in the two groups were comparable (*P* > 0.05). The postoperative radiographic data, the Bohler’s angle, Gissane’s angle, and calcaneal height, width, and length in the two groups were comparable (*P* > 0.05).

**Conclusions:**

Compared with conventional ORIF, the advantages of MIPO are a considerably shortened operating time and hospital stay, decreased postoperative pain, and reduced risk of wound healing complications.

## Background

Calcaneal fractures are the most common fractures of the tarsal bones, constituting 65% of all tarsal fractures and 1–2% of fractures overall [1]. Approximately 70% of calcaneal fractures are intraarticular fractures [2, 3]. Although the treatment of calcaneal fractures remains controversial [4], it has been reported that nonoperative treatment for displaced intraarticular calcaneal fractures has been associated with poor results secondary to subtalar arthrosis and abnormal hindfoot morphology [5]. Thus, many authors advocate for surgical treatment over conservative treatment for these fractures [5, 6]. Since the mid-1990s, open reduction and internal fixation via an extended L-shaped lateral approach has been considered the gold standard for surgical treatment of displaced intraarticular calcaneal fractures [5, 7]. However, postoperative wound healing complications, including wound infection, skin edge necrosis, wound breakdown, and hematoma, remain a major concern [8, 9]. The incidence of wound complications when using extended L-shaped lateral approaches has been reported to be anywhere from 5.8 to 43% [6, 10, 11].

To minimize the incidence of wound complications, several minimally invasive techniques have been developed in recent years, including closed reduction and percutaneous screw fixation [12–14], open reduction via a less invasive sinus tarsi approach [12, 14, 15], and arthroscopic-assisted fixation [12, 16]. Closed reduction and percutaneous screw fixation has frequently been reported and can effectively reduce the risk of operative wound complications as a result of the small incision [12]. However, the screw fixation is not strong enough to offer rigid stabilization of the calcaneal fracture fragments. Additionally, open reduction using a less invasive sinus tarsi approach is recommended by some experts and has the advantages of effective reduction of the subtalar articular surface and greatly decreased impact on local skin blood supply [14]. However, the application of the common calcaneal plate is limited as specially designed plates, which have not undergone biomechanical testing, are applied. More recently, early reports of arthroscopically assisted fixation have been published [12, 16], but a number of factors have prevented it from gaining widespread use, including a steep learning curve with prolonged surgical duration and a costly equipment requirement.

We developed a novel minimally invasive percutaneous osteosynthesis (MIPO) technique for the treatment of displaced intraarticular calcaneal fractures that showed favorable outcomes in our clinical series. In the MIPO technique, a small vertical incision was placed midway between the posterior border of the fibula and the anterolateral border of the Achilles tendon, and percutaneous internal fixation was performed. The purpose of this study was to describe this MIPO technique and to evaluate the clinical outcomes and complications of this technique compared with conventional treatment for Sanders type II and III calcaneal fractures using an extended L-shaped lateral approach.

## Methods

### Study design

This was a prospective randomized controlled trial of patients with displaced intraarticular fractures of the calcaneus. The study was conducted according to the “CONSORT statement” guidelines for randomized controlled trials [17]. Patients were allocated in accordance with the random number table and divided into two groups, a MIPO group and an open reduction and internal fixation (ORIF) using a conventional approach group (conventional treatment group). A calcaneus plate (AO Synthes, Oberdorf, Switzerland) was used for internal fixation in both groups. The procedures were all performed by a team of two senior surgeons. All radiographic assessments were conducted by two independent senior radiologists. The final result was the average of the two testing values. The study protocol was approved by the Medical Ethics Committee of Shaoxing People’s Hospital (No. 2010024).

### Patients

**Fig. 1:**
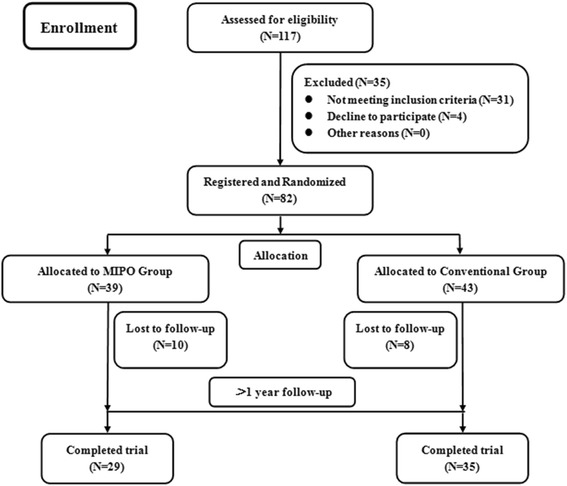
Flow of patients through the study

The inclusion criteria for this study were displaced intraarticular fractures of the calcaneum with standard radiographic and CT evidence of Sanders type II or III fractures. Patients with an open fracture, bilateral calcaneal fractures, peripheral vascular disease, skin infection, signs of compartment syndrome, neurologic deficit following head injury or spinal injury, other fractures of the ipsilateral or contralateral limb, and severe osteoporosis were excluded. From May 2011 to November 2013, 82 consecutive patients with unilateral calcaneal fractures that met these criteria were included in the study. All patients received the type of treatment to which they had been allocated (Fig. 1). Written informed consent was obtained from all participants. Demographic data including age, sex, injury mechanism, and follow-up time were recorded.

### Surgical techniques

Following admission, the patients’ extremities were elevated and ice was applied in an effort to minimize swelling and avoid blisters. Surgery was performed either within 24 h or after the swelling had subsided and skin wrinkling had appeared. For antibiotic prophylaxis, 1.5 g of intravenous cefuroxime was administered 30 min preoperatively [18]. All patients underwent general anesthesia or epidural anesthesia.

**Fig. 2:**
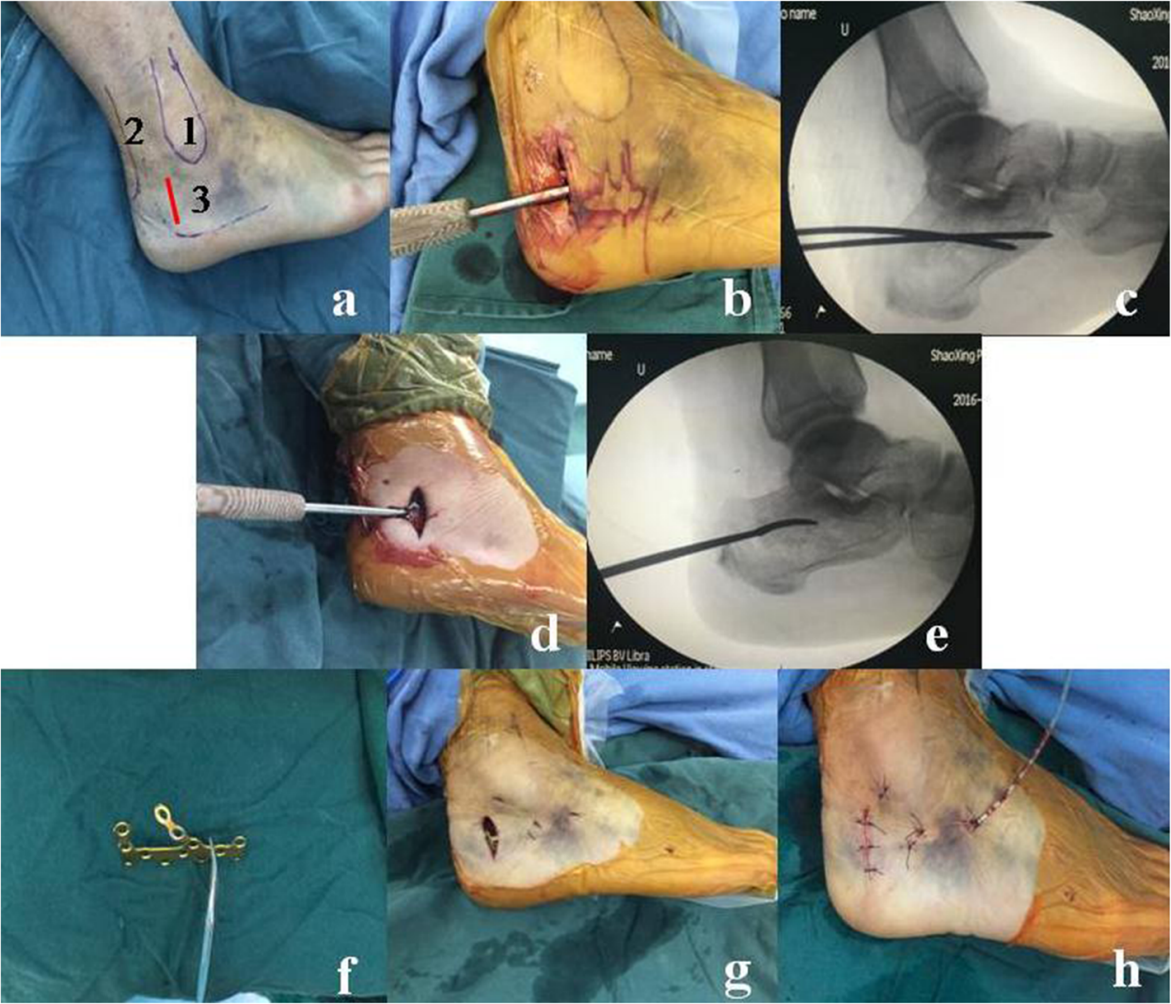
**a** Landmarks for skin incisions were identified *1*) the lateral malleolus, *2*) the Achilles tendon, and *3*) the incision for the MIPO group. **b** A periosteum elevator was used to create a lateral calcaneus subfascial channel. **c** Satisfactory anatomical reduction of the calcaneum is achieved with a Steinmann pin under C-arm fluoroscopy intraoperatively. **d** A periosteum elevator was inserted in the calcaneum and placed under the posterior articular surface. **e** Under C-arm fluoroscopy, the posterior articular surface was directly reduced by leverage with a periosteum elevator. **f** A pre-bent calcaneus plate was prepared. **g** The plate and screw were secured into place. **h** The rubber drains were inserted and the incision was closed in a layered fashion

In the MIPO group, the patient was placed in a lateral position on a radiolucent operating table and a tourniquet was applied. Landmarks for skin incision were identified, including the posterior border of the lateral malleolus and the anterolateral border of the Achilles tendon. An axially directed incision (3–4 cm) was made from the level of the tip of the lateral malleolus to midway between the posterior border of the fibula and the anterolateral border of the Achilles tendon (Fig. 2a). After dividing the subcutaneous tissue directly to the bone, a periosteum elevator was used to create a lateral calcaneal subfascial channel to fit the calcaneal plate (Fig. 2b). Afterward, a Steinmann pin was inserted into the calcaneal tuberosity from the lateral side and traction was applied along the long axis of the foot to correct the varus and length of calcaneus while the posterior articular surface was reduced by leverage (Fig. 2c). For cases of Sanders type III calcaneal fractures, the posterior articular surface was also reduced with the help of a periosteum elevator. After incompletely uncovering the fragment of the lateral wall, a periosteum elevator was inserted in the calcaneus and placed under the posterior articular surface (Fig. 2d). Under C-arm fluoroscopy, the posterior articular surface was directly reduced by leverage (Fig. 2e). Once satisfactory anatomical reduction of the calcaneus was achieved, a pre-bent calcaneus plate (AO Synthes, Oberdorf, Switzerland) (Fig. 2f) was inserted into the subcutaneous calcaneus lateral channel. After confirming the proper placement of the plate under C-arm fluoroscopy, an identical plate was placed on the implant area as a scope for selective stab incisions and screws were placed into the rear calcaneal body, the subtalar articular surface, and the anterior process of the calcaneus through the stab incisions (Fig. 2g). Next, the plate and screw were secured under C-arm fluoroscopy, rubber drains were inserted, and the incision was closed in a layered fashion followed by compression bandaging (Fig. 2h).

In the conventional treatment group, ORIF using an extended L-shaped lateral approach [2] was performed. An incision measuring 10–12 cm was made directly to the bone to create a full-thickness flap. In contrast to the MIPO group, the entire lateral wall of the calcaneus was exposed and the subtalar joints were directly visualized after overturning the fracture fragment of the lateral wall. Once anatomical reduction of the calcaneus was achieved, the allogeneic bone was used for the reconstruction of calcaneal defects. Rigid fixation with the calcaneus plate and screws (AO Synthes, Oberdorf, Switzerland) was also achieved under C-arm fluoroscopy.

Postoperatively, the affected limbs of the patients were elevated to minimize swelling. At 3 and 12 h postoperatively, 1.5 g of prophylactic intravenous cefuroxime was administered [18]. At 24 h postoperatively, it was possible to begin passive and active motion of the ankle and subtalar joint without weight-bearing. Rubber drainage was extracted after 48 h and the incisions were cleaned every 2 days until the sutures were removed approximately 2 weeks after surgery. All patients were treated with analgesic therapy for 1 week (celecoxib 200 mg, orally, twice daily). Partial weight-bearing was begun after 6 weeks post-operation, and full weight-bearing was initiated after 12 weeks.

### Outcome parameters

Primary clinical outcomes including operative time, postoperative visual analog scale (VAS) score, and wound healing complications were recorded. VAS was used to track patients’ daily pain scores during the pre-operation and post-operation phases, as well as at 1 week postoperatively. The VAS was measured in centimeters (0–10.0 cm) in this study. The wound healing complications, including superficial infection, deep infection, and wound necrosis, were evaluated. According to the surgical site infection criteria [19], superficial infections could be treated non-operatively and infections that required surgical intervention, readmission, or intravenous antibiotics were classified as deep infections.

Secondary clinical outcomes including time to operation, length of incision, postoperative drainage, length of hospital stay, and medical expense were also recorded. The postoperative complications of peroneal tendon or sural nerve injury were also assessed. At the final follow-up visit, the American Orthopedic Foot and Ankle Society (AOFAS) Ankle-Hindfoot scale [20] and the Short Form-36 (SF-36) questionnaires [21] were used to quantify the functional outcomes.

**Fig. 3:**
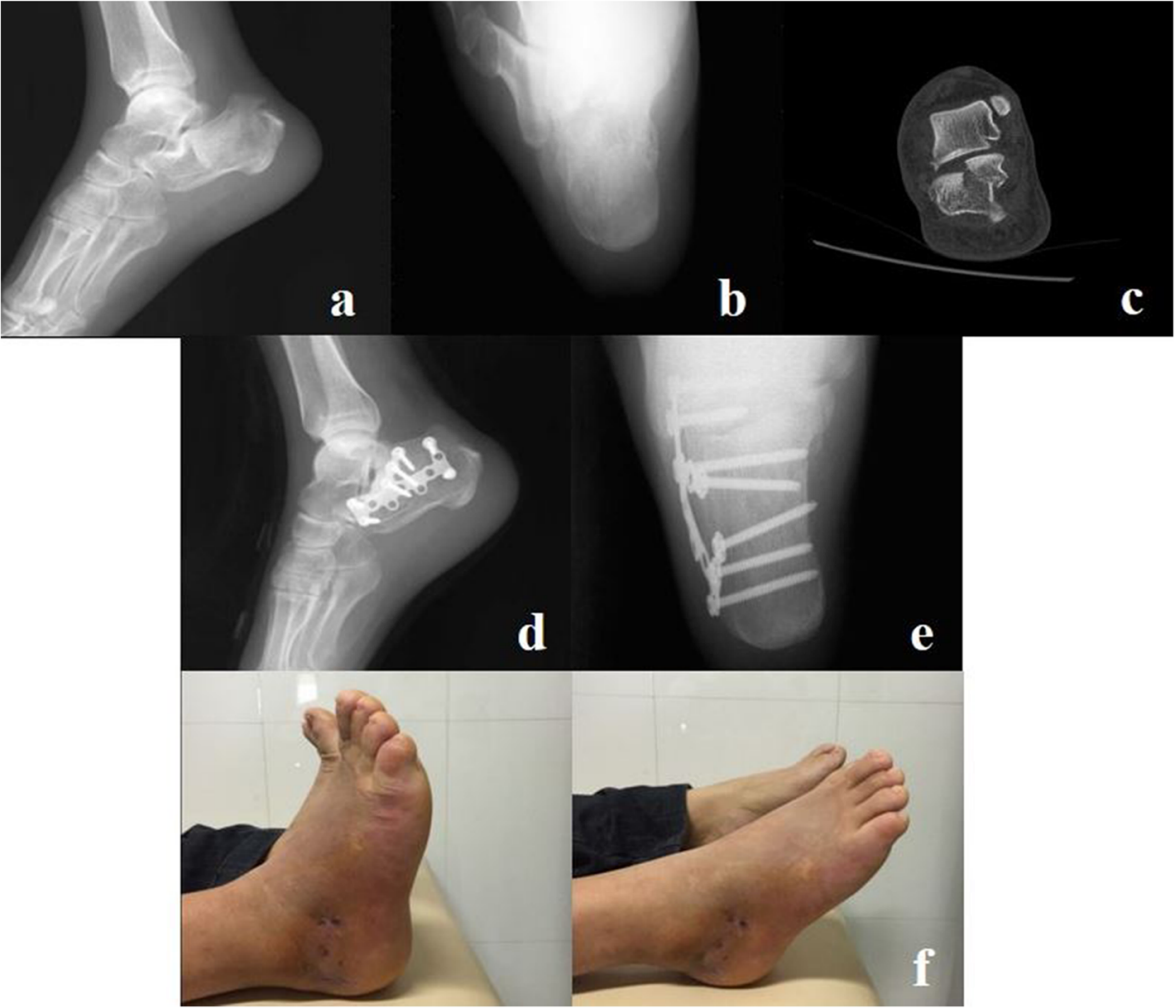
A 36-year-old male patient had a right calcaneal fracture as a result of an accidental fall. **a** Preoperative lateral radiography. **b** Preoperative axial radiography. **c** Preoperatively, the CT scan indicated a Sanders type II fracture. **d** Postoperative lateral radiography. **e** Postoperative axial radiography. **f** Two weeks postoperatively, the motion of the ankle and subtalar joints was almost identical to that of the contralateral limb

Radiographic evaluation included measurement of the height and width of the calcaneus in ankle axial view, Bohler’s angle, Gissane’s angle, and length of the calcaneus on the lateral view during the pre-operation and post-operation period [9, 13–15]. In addition, the surgical planning and classification of fractures using the Sanders et al. [22] classifications were determined by preoperative CT scans (Fig. 3).

### Statistical analysis

Data were expressed as means ± standard deviation. Statistical evaluation including the Pearson *χ* ^2^ test, contingency table *χ* ^2^ test, and Student’s *t* test was conducted for comparisons between the MIPO group and the conventional treatment group. The tests were performed using SPSS 18.0 for Windows. The Shapiro-Wilk and Levene’s tests showed normal data distribution and variance. The level of significance was set at *P* < 0.05.

## Results

### Demographic data

From May 2011 to November 2013, a total of 82 patients who fulfilled the study criteria were initially enrolled; 18 patients were lost to follow-up. The reasons for loss of follow-up included limited activity in 8 cases, loss of communication in 4 cases, and immigration to another area in 6 cases. Of the remaining 64 patients available for analysis, 29 patients had displaced intraarticular calcaneal fractures in the MIPO group and 35 in the conventional treatment group. All patients completed at least 1 year of follow-up. There were no statistically significant differences between the MIPO and conventional treatment groups in terms of age, sex, mechanism of injury, Sanders classification of calcaneal fractures, or follow-up time (Table 1).

**Table 1:** Demographic information

	MIPO group	Conventional group	*P*
Number	29	35	
Sex (M/F)	12/17	19/16	0.304
Age (years)	40.1 ± 12.1	43.6 ± 12.4	0.258
Mechanism of injury			
Traffic injuries	6	4	0.597
Fall	20	27	
Other trauma	3	4	
Sanders classification			
II	18	22	0.948
III	11	13	
Follow-up time (months)	16.9 ± 3.7	15.7 ± 3.5	0.199

### Primary clinical outcomes

The MIPO group was superior to the conventional group in terms of operative time and postoperative VAS values. The operative time of the MIPO group was significantly shorter than that of the conventional treatment group (52.5 ± 11.1 vs. 82.8 ± 16.2 min, respectively; *P* < 0.05). At 1 week postoperatively, the VAS values in the MIPO group (3.2 ± 1.4) were significantly lower than those in the conventional treatment group (3.9 ± 1.3, *P* < 0.05) (Table 2).

**Table 2:** Clinical outcomes

	Group	MIPO group	Conventional group	*P*
Primary	Operative time (min)	52.5 ± 11.1	82.8 ± 16.2	<0.001
	VAS			
	Post-operation	6.9 ± 1.2	6.7 ± 1.4	0.568
	1 week	3.2 ± 1.4	3.9 ± 1.3	0.038
	Wound healing complications	2	13	0.004
	Superficial infection	1	4	0.236
	Deep infection	0	2	0.191
	Wound necrosis	1	7	0.046
Secondary	Time to operation (days)	6.2 ± 3.1	6.5 ± 2.7	0.647
	Length of incision	4.2 ± 0.6	10.9 ± 1.5	<0.001
	Postoperative drainage (mL)	169.5 ± 49.1	160.7 ± 43.9	0.457
	Hospital stay (days)	9.7 ± 2.8	11.7 ± 2.6	0.004
	Medical expense (10^2^ USD)	20.0 ± 2.3	21.3 ± 4.4	0.143
	AOFAS score	84.4 ± 4.9	83.9 ± 6.2	0.672
	SF-36 score	83.9 ± 5.3	84.8 ± 5.9	0.533

Overall, the incidence of wound healing complications in the MIPO group was significantly lower than that of the conventional treatment group. In the conventional treatment group, 13 of 35 fractures (37.1%) had issues with wound healing compared to only 2 of 29 fractures (6.7%) in the MIPO group; this difference was statistically significant (*P* < 0.05). Deep infection occurred in none of the 29 patients in the MIPO group and in 2 of the 35 (5.7%) patients in the conventional treatment group (*P* = 0.191). The incidence of superficial infection was 1 of 29 (3.4%) patients in the MIPO group and 4 of 35 (11.4%) patients in the conventional treatment group (*P* = 0.236). Wound necrosis occurred in 1 of 29 (3.4%) patients in the MIPO group and 7 of 35 (20%) patients in the conventional treatment group, and this difference was statistically significant (Table 2). Five patients with superficial infections in the two groups were managed using dressings and oral antibiotics. Two patients with deep infections were treated with debridement twice as well as with intravenous antibiotics, without success. Finally, implant removal was executed.

### Secondary clinical outcomes

The length of hospital stay was 9.7 ± 2.8 days in the MIPO group and 11.7 ± 2.6 days in the conventional treatment group (*P* < 0.05). The length of incision was 4.2 ± 0.6 cm in the MIPO group and 10.9 ± 1.5 cm in the conventional treatment group (*P* < 0.05). Neither peroneal tendon nor sural nerve injury occurred in any patient in either group. The medical cost was 20.0 ± 2.3 × 10^2^ USD in the MIPO group and 21.3 ± 4.4 × 10^2^ USD in the conventional treatment group, with no statistical difference. In terms of time to operation and postoperative drainage, the data were comparable between the two groups (*P* > 0.05). At the time of the final follow-up, the SF-36 scores and AOFAS scores in the two groups were comparable (*P* > 0.05) (Table 2).

### Radiographic outcomes

Similar radiographic results were achieved in both groups. Preoperatively, all the radiographic outcomes in the two groups were comparable (*P* > 0.05). Postoperative radiography showed that satisfactory calcaneal fracture reduction was achieved in both groups. The postoperative Bohler’s angle was 28.7 ± 4.6° in the MIPO group and 29.5 ± 4.1° in the conventional treatment group (*P* > 0.05). The postoperative Gissane’s angle was 123.0 ± 10.5° in the MIPO group, which was lower than that in the conventional treatment group, 124.3 ± 10.1°, but the difference was not statistically significant (*P* = 0.620). There were no significant differences in the calcaneal height, width, or length between the two groups postoperatively (*P* > 0.05) (Table 3).

**Table 3:** Radiographic outcomes

Observation indexes	Pre-operation			Post-operation		
	MIPO group	Conventional group	*P*	MIPO group	Conventional group	*P*
Bohler’s angle(°)	3.7 ± 2.9	3.4 ± 2.4	0.709	28.7 ± 4.6	29.5 ± 4.1	0.473
Gissane’s angle(°)	93.0 ± 10.2	91.1 ± 10.1	0.442	123.0 ± 10.5	124.3 ± 10.1	0.620
Height(mm)	32.2 ± 4.1	33.1 ± 3.8	0.332	37.9 ± 3.0	38.5 ± 3.7	0.473
Width(mm)	39.4 ± 4.2	39.8 ± 3.7	0.707	34.2 ± 3.1	33.9 ± 3.0	0.679
Length(mm)	62.7 ± 7.9	63.4 ± 5.5	0.613	67.6 ± 5.3	68.7 ± 5.0	0.365

## Discussion

The foremost benefit of MIPO is the reduced risk of wound healing complications. The entire blood supply of the lateral skin is derived from the peroneal artery and its terminal branches. The L-shaped lateral incision must specifically avoid this artery and its branches, and it will result in a severe risk of wound necrosis and soft tissue infection [23]. In addition, the lateral wall of the calcaneus is subcutaneous and any implants on the surface can lead to wound healing complications [13]. Thus, the risk of postoperative wound healing complications using the extended L-shaped lateral approach is quite high; in fact, Buckley et al. reported a 25% rate of wound healing issues [6]. Likewise, DeWall et al. reported a deep infection rate of 14.3% and superficial infection rate of 21.4% in patients treated with open reduction using an extended L-shaped lateral approach [24]. Similar to the previous studies, 13 of the 35 fractures (37.1%) in the conventional treatment group in our study experienced wound healing problems; deep infection occurred in 2 of the 35 (5.7%) fractures and the incidence of superficial infection was 4 in 35 (11.4%). However, only 2 patients (6.7%) in the MIPO group experienced a wound or soft tissue complication or infection. Compared with conventional treatment, MIPO substantially decreased the risk of wound complications because of minimal soft tissue dissection and lateral skin blood supply preservation.

The fact that this technique was minimally invasive was also evidenced by the shortened operating time, decreased postoperative pain, and shortened hospital stay. In our study, the operative time and length of hospital stay in the MIPO group were considerably shorter than those in the conventional treatment group with a statistically significant difference. Additionally, the VAS value in the MIPO group was lower than that of the conventional treatment group at 1 week postoperatively. These data demonstrate that minimal soft tissue dissection by MIPO leads to better postoperative rehabilitation. According to the previous literature, the operative risks of the L-shaped lateral approach include injury to the peroneal tendons and the sural nerve [15]. Although neither of those occurred in the two groups in our study, MIPO generally prevents these complications by employing careful subperiosteal dissection and percutaneous internal fixation.

The allowed application of a common calcaneus plate in MIPO was also an added advantage compared with previous minimally invasive techniques. Closed reduction and percutaneous fixation have been widely reported to significantly reduce the incidence of wound healing complications. De Vroome et al. reported that postoperative infection occurred in 1 of 46 (2.4%) cases treated using closed reduction and percutaneous screw fixation [25]. However, this treatment seems to be more suitable for cases with moderately displaced fracture or non-comminuted calcaneus fracture because of the limited fixation strength. For instance, Stulik et al. reported 13 cases (4.5%) of loss of reduction in patients treated with closed reduction and percutaneous fixation using K-wires [26]. In our study, a common calcaneus plate was applied in the MIPO group which can offer rigid fixation of calcaneal fracture fragments. Minimally invasive sinus tarsi approach was also recommended by some authors due to fewer wound complications and easy subtalar articular reduction. Kline et al. reported the overall rate of wound complications was 6% in the patients treated with minimally invasive sinus tarsi approach [9]. Xia et al. also reported shorter surgical times and lower wound complications using the minimally invasive sinus tarsi approach compared with an extended L-shaped lateral approach [27]. However, compared with our minimally invasive technique, the application of a common calcaneal plate is limited and some specially designed plates, which have not undergone biomechanical testing, have been used in the sinus tarsi approach.

One of the most important aspects of this technique pertains to the skin incision. An axially directed incision (3–4 cm) was made from the level of the tip of the lateral malleolus and placed midway between the posterior border of the fibula and the anterolateral border of the Achilles tendon. This type of small incision greatly decreases the impact on local skin blood supply and the length of the incision depends only on the width of the plate. Additionally, a lateral calcaneus subfascial channel should be created by careful subperiosteal dissection to avoid injury to the peroneal tendons and the sural nerve. Furthermore, an identical plate can be used as a scope to ensure the accuracy and precision of the screw placement.

A satisfactory anatomical reduction was also achieved by this minimally invasive technique. Although the use of MIPO to treat calcaneal fractures carries a risk of inadequate reduction, especially in terms of posterior facet reconstruction, we achieved satisfactory anatomical reduction of the calcaneum in the two groups in our study. Additionally, we did not use bone grafting in the MIPO group and allogeneic bone was used to fill the calcaneal defect in the conventional treatment group. While bone grafts do add mechanical support and may stimulate quicker fracture healing, the calcaneus is extremely vascular and heals rapidly with reconstitution of the cancellous bone within 4 to 8 weeks of surgery [13]. Furthermore, the possibility that the use of bone substitutes increases the risk of infection is a definite source of concern [28].

This study had several limitations of note. First, we had a relatively small number of patients and a poor power analysis (*β* < 0.8) was calculated in several primary clinical outcome parameters. In addition, we had a mean follow-up period of only 14 months; a longer follow-up period is needed to adequately assess the arthritic changes in the subtalar joint, which are among the most common complications. For instance, Thermann et al. reported arthritic changes in the subtalar joint in 65.2% of type II fractures and in 81.7% of type III and type IV fractures and radiological follow-up evaluation showed arthritic changes in the subtalar joint in 44% of cases [29]. Furthermore, we achieved satisfactory anatomical reduction of the calcaneus in the two groups in this study; however, the quality of fracture reduction was not assessed in this study. At the last follow-up, radiographic evaluation was not routinely underwent in majority of patients on account of the medical cost, and the radiological outcomes of the last follow-up were also not showed in this study. Lastly, we had a significant loss to follow-up as 18 (21.20%) patients did not return to the clinic and thus did not complete the questionnaires. Therefore, further investigation with a larger sample size and a longer follow-up period is necessary to obtain a more precise understanding of the clinical efficacy of MIPO in the management of intraarticular calcaneal fractures.

## Conclusions

From our experience with the 29 patients in the MIPO group, we believe that the radiologic and clinical results achieved by our patients were satisfactory. Compared with the conventional treatment using an L-shaped lateral approach for intraarticular calcaneus fractures, MIPO offers critical advantages, including a considerably reduced risk of wound healing complications, shortened operating time and length of hospital stay, as well as superior postoperative rehabilitation.

## Abbreviations

AOFAS: American Orthopedic Foot and Ankle Society
MIPO: Minimally invasive percutaneous osteosynthesis
ORIF: Open reduction and internal fixation
SF-36: Short Form-36
USD: United States dollar
VAS: Visual analog scale
